# Pathophysiology of Methicillin-Resistant *Staphylococcus aureus* Superinfection in COVID-19 Patients

**DOI:** 10.3390/pathophysiology29030032

**Published:** 2022-07-27

**Authors:** Gul Habib, Khalid Mahmood, Haji Gul, Muhammad Tariq, Qurat Ul Ain, Azam Hayat, Mujaddad Ur Rehman

**Affiliations:** 1 Department of Microbiology, Abbottabad University of Science and Technology, Havelian, Abbottabad 22010, Pakistan; quratulain1998.qu@gmail.com (Q.U.A.); azam@aust.edu.pk (A.H.); mujaddad@aust.edu.pk (M.U.R.); 2 National Institute of Virology, Dr. Panjwani Center for Molecular Medicine and Drug Research, International Center for Chemical and Biological Sciences, University of Karachi, Karachi 75850, Pakistan; qureshi.qau@gmail.com; 3 College of Animal Science and Technology, Anhui Agricultural University, Hefei 230036, China; hbb.scilab@gmail.com; 4 Institute of Paramedical Sciences, Khyber Medical University, Peshawar 25000, Pakistan; microbiologylab23@gmail.com

**Keywords:** MRSA, COVID-19, *mecA*, antibiotic resistance, pneumonia

## Abstract

The global spread of the coronavirus disease 2019 (COVID-19), caused by severe acute respiratory syndrome coronavirus-2 (SARS-CoV-2), has infected humans in all age groups, deteriorated host immune responses, and caused millions of deaths. The reasons for individuals succumbing to COVID-19 were not only the SARS-CoV-2 infection but also associated bacterial infections. Antibiotics were largely used to prevent bacterial infections during COVID-19 illness, and many bacteria became resistant to conventional antibiotics. Although COVID-19 was considered the main culprit behind the millions of deaths, bacterial coinfections and superinfections were the major factors that increased the mortality rate in hospitalized patients. In the present study, we assessed the pathophysiology of methicillin-resistant *Staphylococcus aureus* (MRSA) superinfection in COVID-19 patients in Pakistan. A total of 3492 COVID-19 hospitalized patients were screened among which 224 strain were resistant to methicillin; 110 strains were tazobactam-resistant; 53 strains were ciprofloxacin-resistant; 23 strains were gentamicin-resistant; 11 strains were azithromycin-resistant; 3 strains were vancomycin-resistant. A high frequency of MRSA was detected in patients aged ≥50 with a prevalence of 7.33%, followed by patients aged >65 with a prevalence of 5.48% and a 5.10% prevalence in patients aged <50. In addition, pneumonia was detected in the COVID-19-associated MRSA (COVID-MRSA) that showed decreased levels of lymphocytes and albumin and increased the mortality rate from 2.3% to 25.23%. Collectively, an MRSA superinfection was associated with increased mortality in COVID-19 after 12 to 18 days of hospitalization. The study assessed the prevalence of MRSA, mortality rate, pneumonia, and the emergence of antibiotic resistance as the main outcomes. The study summarized that COVID-MRSA aggravated the treatment and recovery of patients and suggested testing MRSA as critical for hospitalized patients.

## 1. Introduction

SARS-CoV-2 is a positive, single-stranded RNA virus of the family coronaviridae and has caused severe morbidity and mortality in the elderly population, immunocompromised individuals, and patients with comorbidities [1]. These individuals were highly vulnerable to hospital and community-associated bacterial infection during the COVID-19 pandemic. Bacterial infections associated with COVID-19 can be coinfections or superinfections. Based on the Center for Disease Control and Prevention (US) definition, bacterial coinfection is the concurrent infection with the initial infection, which means that coinfection takes place simultaneously, while superinfections are the types of infections that follow on a previous infection. Superinfections develop after initial infections and are caused by resistant bacteria or bacteria that acquired resistance to antibiotics used earlier. In fact, these infections increased the morbidity and mortality rates during past pandemics; for instance, the Spanish influenza pandemic was caused by the H1N1 virus that caused approximately 50–100 million deaths. The major reason for death was thought to be the secondary bacterial pneumonia but not the H1N1 virus itself. Similarly, in the 2009 swine flu pandemic, bacterial superinfections accounted for more than 50% of deaths [2,3]. SARS-CoV-2 has been widely studied during the pandemic and has impacted the social and medical fields. Though COVID-19 was the main reason behind the death of more than 6 million people across the globe, some known pathogens that contributed to the severity of COVID-19 remained unnoticed during the different waves of the pandemic [4]. During COVID-19 treatment, multiple antibiotics were used to counter bacterial infections, such as ampicillin, co-amoxiclav, tazobactam, ciprofloxacin, azithromycin, etc. Due to less focus on antibiotic-resistant bacteria during the pandemic, bacterial infections worsened COVID-19 prognosis and treatment. Hence, the emergence of antibiotic-resistant bacteria was not impracticable, and a large number of patients were infected with different bacteria, and resistant strains were reported [5]. *S. aureus* colonizes the skin, nose, and throat of humans and is associated with community- and hospital-associated infections. *S. aureus* infection is common in immunocompromised patients, intensive care unit patients, pneumonia cases, and viral infections, and MRSA strains were widely reported from these patients [6,7,8]. The guidelines of the Infectious Diseases Society of America recommended the use of anti-MRSA agents and protective measures for critically ill patients during hospitalization [1,9,10]. Bacterial infection with COVID-19 was reported in the USA [11], UK [12], China [13], Saudi Arabia [14], and other countries as well [15,16]. Pakistan had reported more than 15 million positive cases and 0.3 million deaths by May 30, 2022. In our previous work, we reported the COVID-19 mortality and morbidity details and discovered that a persistent high ambient temperature with supportive environmental factors was crucial in mitigating the COVID-19 epidemic in Pakistan [17]. Here, for the first time, we reported MRSA superinfections among patients infected with COVID-19 from Pakistan and detected 224 positive COVID-MRSA cases. Recently, *Clostridium difficile* [18], *Enterobacteriaceae*, and *S. aureus* coinfections have been reported [19], but an MRSA superinfection has not been investigated thoroughly [20,21]. This study highlighted the course of hospitalization, antibiotic resistance development, detection of pneumonia in COVID-MRSA, and MRSA mortality rate in COVID-19 patients.

## 2. Materials and Methods

This was an experimental study that involved a large screening of bacterial isolates from COVID-19 patients between the years 2020 and 2021. The clinical data of symptomatic patients who tested positive for SARS-CoV-2 by quantitative reverse transcription-polymerase chain reaction (RT-qPCR) were included from the hospitals of Khyber-Pakhtunkhwa and Sindh. The data collected include epidemiological and demographic findings, signs and symptoms during hospitalization, and antibiotic resistance profiling. The prognosis of bacterial superinfection was performed by the stay at the hospital (hours ≥ 48), the severity of the disease, clinical symptoms, and blood biomarker testing. Laboratory biomarker tests, such as C-reactive protein (CRP), erythrocyte sedimentation rate (ESR), lactate dehydrogenase (LDH), creatine kinase (CK), alanine aminotransferase (ALT), alkaline phosphatase (ALP), white blood cell (WBC), red blood cell (RBC), D-dimer, ferritin, serum creatinine, troponin-I, and immune cell counting, were performed by the hospital in question on the day of admission and monitored until recovered/diseased. The prevalence of MRSA was calculated from the day of admission to 25 days of hospitalization. Nasopharyngeal and endotracheal aspirate specimens were collected from patients and were transferred to a bacteriological laboratory. The samples were cultured on tryptic soy agar (Sigma-Aldrich, Darmstadt, Germany), mannitol salt agar (Sigma-Aldrich Darmstadt, Germany), and MacConkey agar (Sigma-Aldrich, Darmstadt, Germany) and were incubated at 37 °C for 24–48 h. Then, we performed antibiotic sensitivity testing and selected the highly resistant isolates. Further, we determined the minimum inhibitory concentration (MIC) according to the protocol of the Clinical and Laboratory Standards Institute [22]. The antibiotic susceptibility assay was performed as previously described [8]. The methicillin-resistant isolates were cultured in tryptic soy broth (TSB), and the genome was extracted by Thermo Scientific GeneJET Genomic DNA Purification Kit (Thermo Fisher, Waltham, MA, USA). The *mecA* gene was amplified (700 bp fragment) from resistant strains using the following primers: *mecA*-forward-1 ATGAGAATAGAACGAGTAGA and *mecA*-reversed-2 TTATTCAGTTGTCTCTGGAA.

### 2.1. RNA Extraction and RT-qPCR 

The COVID-19 sample RNA was extracted by TANBead nucleic acid extraction kit (Taiwan Advanced Nanotech Inc., Taoyuan, Taiwan). The genesig RT-PCR coronavirus kit (Primerdesign Ltd., Chandler’s Ford, UK) was used for the detection of viral RNA. The PCR program (55 °C for 10 min, 95 °C for 2 min, 45 cycles of 95 °C for 10 s, and 60 °C for 60 s) was run by the Agilent AriaMx Real Time-PCR G-8830 system (Santa Clara, CA, USA) according to the kit manufacturer’s instructions. For *mecA* gene amplification, Prime Star PCR Kit (TaKaRa Tokyo, Japan) was used with the following PCR program (95 °C for 5 min, 95 °C for 15 s, 55 °C for 35 s, 72 °C for 1 min, 30 cycles, and 72 °C for 10 min).

### 2.2. Data Analysis

The continuous variables were presented as median (interquartile range) and compared by the Mann–Whitney test. The categorical variables were presented as percentages and compared by the Chi-square test. The Wilcoxon test was used for survival curve plotting, and the association between superinfection and mortality was analyzed by Pearson correlation and log-rank Mantel–Cox tests. Graphpad Prism 8 was used for data analysis. All analysis with *p* < 0.05 was considered significant. 

### 2.3. Ethical Approval

The study was approved by Ethical Review Committee of the Department of Microbiology, Abbottabad University of Science and Technology (Approval Code: AUST/ORIC/2021/375; Approval Date: 29 September 2021). All patient data were deidentified, and informed consent was waived.

## 3. Results

**Figure 1:**
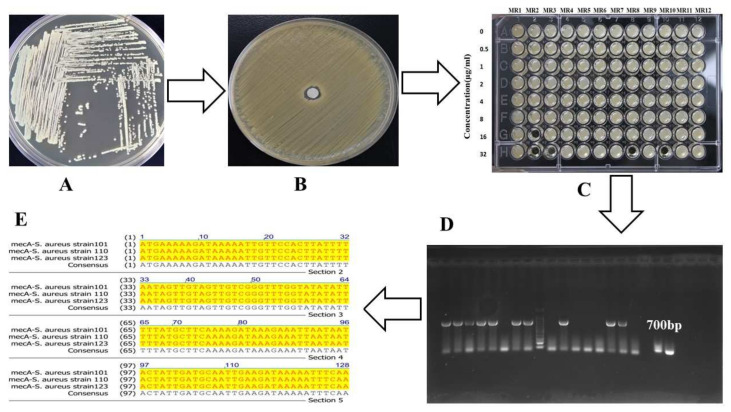
This figure summarizes the schematic diagram of screening resistant isolates from COVID-19 patients. (**A**) A single colony was streaked on TSA plate for antibiotic sensitivity testing. (**B**) The isolated strain antibiotic sensitivity testing was performed on Mueller–Hinton agar, and methicillin-resistant strains were selected. (**C**) The isolated strains MIC against methicillin, tazobactam, ciprofloxacin, etc. were determined by two-fold broth microdilution method ranges from 0 to 32 µg/mL and higher (512 µg/mL), while MR1–MR12 represent different strains. (**D**) *mecA* gene (700 bp fragment) was amplified from resistant isolates by PCR, and the product bands were visualized by gel electrophoresis, and (**E**) the PCR purified product of *mecA* gene (700 bp) was sequenced to confirm *mecA* detection.

We screened the COVID-19 samples in three steps: initially, 3492 COVID-19 positive samples were selected with variant levels of LDH, CRP, D-dimer, lymphocytes, AST, ALT, CK, albumin, and procalcitonin compared to the reference values. The selected samples were screened for bacteria isolation and cultured on different media, such as tryptic soy agar, mannitol salt agar, and MacConkey agar, and methicillin, tazobactam, ciprofloxacin, gentamicin, and vancomycin sensitivity testing were performed. Further, MIC was determined, and methicillin-resistant (≥32 µg/mL) strains were selected. The methicillin-resistant strain genomes were extracted, and a 700 bp fragment of *mecA* was amplified by PCR and confirmed by sequencing (Figure 1). 

Among 3492 samples, 214 were MRSA, 110 were azithromycin-resistant, 53 were ciprofloxacin-resistant, 23 were gentamicin-resistant, 11 were azithromycin-resistant, 3 were vancomycin-resistant, and 118 cases were multiple antibiotic-resistant as shown in Table 1. The highest number of MRSA cases (102) were detected in patients aged ≥50; 68 cases were detected in patients aged >65, and 44 cases were detected in patients aged <50 (Table 1). 

**Figure 2:**
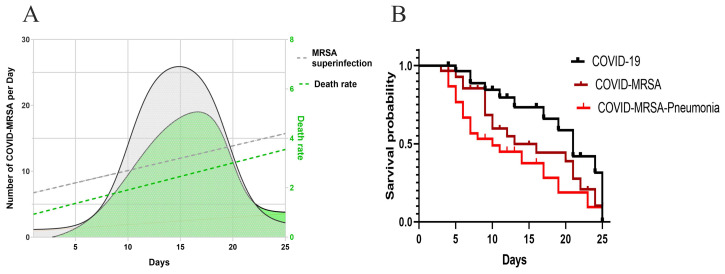
(**A**) The data of COVID-MRSA superinfection and death rate revealed that the MRSA superinfection was evident after 12 days, while the mortality rate increased after 10 days of hospitalization. The Wilcoxon and Spearman correlation analyses showed a significant association between continuous variable and hospitalization days (r = 0.94; *p* < 0.001). (**B**) The black curve indicates COVID-19 infection; the orange curve displays COVID-MRSA, and the red curve shows COVID-MRSA pneumonia. The survival comparison is shown among three states of COVID-19 that revealed those patients with COVID-MRSA pneumonia were subjected to a higher death rate (*p* < 0.01).

The prevalence of MRSA in COVID-19 patients in the early days (1–7) was 3.90%, after 14 days 9.72%, and after 25 days 5.77%, which indicated MRSA superinfection in hospitalized patients. The *mecA* gene was predominantly detected in 214 out of 224 strains with specific primers (*mecA*-forward-1 ATGAGAATAGAACGAGTAGA and *mecA*-reversed-2 TTATTCAGTTGTCTCTGGAA) that confirmed MRSA detection. The number of MRSA superinfections and mortality rate significantly increased (*p* < 0.01) after 12 days of stay, while a decrease (*p* < 0.05) was witnessed after 18 days of hospitalization that designated hospital-acquired MRSA superinfections in COVID-19 patients (Figure 2A). The lowest survival probability was detected in COVID-MRSA pneumonia (*p* < 0.01), compared to COVID-MRSA and COVID-19 as shown in Figure 2B. 

Apart from typical clinical characteristics of COVID-19, COVID-MRSA showed rapid breathing, severe chest pain, cough with phlegm, and patients needed intensive care unit admission more frequently than COVID-19. There were no differences between the groups in the typical COVID-19 symptoms except COVID-MRSA patients showed chronic pneumonia. We detected the signs and symptoms of bacterial pneumonia in 286 antibiotic resistance cases (Table 1) and compared them with COIVD-19 patients without pneumonia. Bacterial pneumonia had the presence of productive cough (phlegm/mucus), whereas COVID-19 had a dry cough. Most of the COVID-19 patients did not have sputum expectoration, but some had mucoid sputum with a white appearance, whereas bacterial pneumonia patients had purulent sputum with a darker yellow or greenish tinge. From radiologic diagnosis, the COVID-19 patients caused multiple peripheral consolidations and glassy opacities, whereas bacterial pneumonia depicted single foci and air bronchograms with a fuzzy appearance. Further, we grouped the blood biomarkers in conjunction with clinical signs and symptoms of COVID-19 with bacterial pneumonia and compared them to patients without pneumonia. A detailed comparison of laboratory biomarkers between COVID-19 patients with and without pneumonia indicated an increased level of neutrophil, procalcitonin, ESR, CRP, AST, D-dimer, LDH, severe cough, chest pain, and severe sweating in pneumonia patients (Table 2). 

COVID-19 patients with pneumonia had decreased levels of lymphocytes and albumin, while ALT, RBC, platelets, monocytes, eosinophils, ferritin, troponin-I, blood urea, and creatinine were not significantly changed compared to COVID-19 patients (Table 2). Collectively, the main risk factors were older age (*p* < 0.05), antibiotic resistance (*p* < 0.01), and bacterial pneumonia (*p* < 0.001) that increased the mortality rate in COVID-MRSA patients.

## 4. Discussion

Coronaviruses are the emerging pathogens that cause epidemics and pandemics worldwide. The data on COVID-19 with bacterial coinfections have been widely reported, but the superinfections have a few reports with a limited number of sample size [23,24]. MRSA is a difficult-to treat-infection and is on the high priority list of the World Health Organization for new antibiotic discovery [7,8,25]. COVID-19 is a respiratory illness, and most of the coinfection was of the lower respiratory tract, thus nasal and oral swabs samples had the highest proportion of *S. aureus* [1,10], while MRSA superinfection occurred in patients hospitalized for a longer period. Bacterial coinfection and superinfection among patients with critical conditions were also reported during the previous epidemics of severe acute respiratory syndrome and Middle East respiratory syndrome [26]. Hospital-acquired pneumonia was reported in 11.5% of COVID-19 cases [13], which is in accordance with our current finding of 8.93% of pneumonia in COVID-MRSA patients. In a study in China, different types of bacterial pathogens were detected in respiratory samples from COVID-19 patients including *Streptococcus pneumoniae*, *Escherichia coli*, *Klebsiella pneumoniae*, *S. aureus*, *Haemophilus influenza* [9], and *Mycoplasma pneumoniae* [21]. A meta-analysis study reported a 5.9% incidence rate of bacterial coinfections and 14.3% of superinfections in COVID-19 patients [20], whereas our results concluded 5.52% MRSA superinfections with a 25.23% mortality rate in hospitalized patients. According to Lansbury et al. 2020 [21], a rate 7% for bacterial infections among hospitalized patients was reported, while a study from India reported 50 cases of coinfections in COVID-19 [27]. A study from Brazil reported *Clostridium difficile* and *E. coli* as the most common bacterial pathogen with prevalence rates of 16.9% and 15.5%, respectively [18], whereas we discovered MRSA infection as being most prevalent in COVID-19 patients during hospitalization. Based on previous reports, lymphocytopenia was common in COVID-19 patients with a normal level of WBC count and increased level of D-dimer, ALT, CK, and LDH [28]. Presently, we witnessed an increased level of neutrophils, procalcitonin, WBC, EST, and AST and a decreased level of albumin in COVID-MRSA pneumonia patients, whereas LDH, D-dimer, ferritin, troponin-I, CK, and CRP were increased in both COVID-MRSA as well as in COVID-19 patients. The increased levels of ESR and CRP indicated inflammatory activity in the body, while an increased level of procalcitonin designated bacterial coinfection because the procalcitonin level increased in pneumonia and other diseases as well [1,9,10]. These biomarker tests have good sensitivity for bacterial infection diagnosis but are less specific in pathogen identification and were useful in distinguishing bacterial superinfection from COVID-19. These blood biomarkers are the indicators of different body organs, and increased levels of LDH, CRP, EST, ALT, AST, CK, procalcitonin, and WBC collectively determined a multiorgan dysfunction. Overall, the COVID-19 mortality rate in Pakistan was 2.3% in the years 2020 to 2021 (https://covid.gov.pk/, accessed on 24 April 2022); [17,29], but the COVID-MRSA showed a 25.23% mortality rate. The reason for such a high mortality might be severe pneumonia, which increased the death rate in COVID-MRSA patients. We found a correlation between MRSA infection with age because COVID-19 patients aged > 50 were more likely to have superinfection; those patients have weaker immunity, and the SARS-CoV-2 mortality rate was high in older patients indicating immune system failure against dual pathogens. Hence, patients with old age, weaker immunity, misuse of antibiotics and immunosuppressants, and pneumonia were at higher risk. The limitation of this study includes that our results were from 3492 positive cases and focused on MRSA only. Secondly, we could not differentiate bacterial pneumonia from viral pneumonia and did not collect details of antibiotics used by patients before hospitalization. This is the first report that comprehensively characterized COVID-MRSA and revealed the clinical outcomes of antibiotic resistance and blood biomarkers that will help in understanding the severity of epidemics and pandemics in the future.

## 5. Conclusions

There is a myriad of data available describing COVID-19 coinfection with *E. coli*, *S. pneumoniae*, *K. pneumoniae*, *H. influenza*, *M. pneumoniae*, *C. difficile*, and *S. aureus*, but limited reports delineated MRSA superinfection. This study concludes that MRSA superinfection increased mortality rate, aggravated patient recovery, and augmented severity of SARS-CoV-2. Considering the high mortality rate with MRSA superinfection, outpatient treatment, vaccination, physician vigilance, proper antibiotic prescription, and testing of MRSA in hospitalized patients are recommended to reduce the risk of superinfections. 

## Figures and Tables

**Table 1:** The prevalence of MRSA and antibiotic resistance in COVID-19 patients. ND; not determined.

Antibiotic Resistance	Number of Isolates	*mecA* Gene Detected	Pneumonia Cases	MIC of *mecA*-Positive Strains (Interquartile Range µg/mL)
Resistance to methicillin	224	159	192	32–512
Resistance to tazobactam	110	36	66	16–128
Resistance to ciprofloxacin	53	4	12	4–16
Resistance to gentamicin	23	3	7	2–16
Resistance to azithromycin	11	6	6	4–32
Resistance to vancomycin	3	2	3	1–4
Resistance to methicillin and tazobactam	68	68	64	ND
Resistance to methicillin and azithromycin	17	6	10	ND
Resistance to tazobactam and azithromycin	26	ND	7	ND
**MRSA patients**	**Age > 65**	**Age ≥ 50**	**Age < 50**	**Total**
7-day hospitalization	21	30	13	64
14-day hospitalization	33	51	22	106
25-day hospitalization	14	21	9	44
Total MRSA positive	68	102	44	214
Total number of patients tested for MRSA	1240	1390	862	3492
COVID-MRSA Prevalence (%)	5.48	7.33	5.10	5.52
Mortality rate (%)	27.9	25.49	20.45	25.23

**Table 2:** Clinical characteristics of COVID-19 patients with and without pneumonia.

Variables (Reference Range)	COVID-19 with Pneumonia	COVID-19	*p*-Value
Body temperature	39–41 °C	38–39.5 °C	
Cough	More	Less	
Chest pain	High	Low	
Breathing problem	High	Low	
Sputum	More	Less	
Sweating	High	Less	
WBC (4–10 × 10^9^/L)	8–15.6 × 10^9^	1.5–8.6 × 10^9^	<0.05
Lymphocytes (1.1–3.2 × 10^9^/L)	0.2–1.0 × 10^9^	0.75–2.1 × 10^9^	<0.05
Neutrophils (45–75%)	55–110	35–80	<0.05
Procalcitonin (<0.15 ng/mL)	0.5–22.0	0.2–6.5	<0.001
Albumin (30–55 mg/L)	10–24	24–40	<0.05
EST (1–20 mm/h)	40–100	30–50	<0.001
AST (15–40 U/L)	45–105	35–75	<0.05
ALT (9–50 U/L)	12–65	15–85	>0.05
LDH (120–250 U/L)	640–1780	300–1050	<0.001
CK (50–310 U/L)	65–460	50–550	<0.05
CRP (0–4 mg/L)	20–130	20–90	<0.05
D-dimer (<200 ng/mL)	600–1800	650–1200	<0.05
RBC (4–6 × 10^6^ cell/µL)	3.0–6.5 × 10^6^	3.0–7.0 × 10^6^	>0.05
Platelets (150–450 × 10^3^/µL)	110–400 × 10^3^	130–450 × 10^3^	>0.05
Monocytes (2–10%)	2–9	1–11	>0.05
Eosinophils (0–0.6%)	0.1–0.7	0.1–0.7	>0.05
Ferritin (30–400 ng/mL)	50–600	60–800	>0.05
Troponin-I (<0.6 ng/mL)	3.5–8.0	5.5–10.5	>0.05
Blood urea (18–45 mg/dL)	15–50	15–50	>0.05
Creatinine (0.5–1.2 mg/dL)	0.5–2.0	0.5–2.0	>0.05

## Data Availability

Not applicable.
